# Electrospun Poly(vinylidene fluoride) Nanocomposites with Ionic Liquid Functionalized Graphene Nanoplatelets by a Noncovalent Method for Piezoresistive Pressure Sensor Applications

**DOI:** 10.1021/acsomega.4c06452

**Published:** 2024-11-05

**Authors:** Lucas Simon, Sébastien Livi, Guilherme M.O. Barra, Claudia Merlini

**Affiliations:** § Mechanical Engineering Department, Federal University of Santa Catarina (UFSC), Florianópolis, SC 88040-900, Brazil; † Université Claude Bernard Lyon 1, INSA Lyon, Université Jean Monnet, CNRS UMR 5223, Ingénierie des Matériaux Polymères, F-69621, Villeurbanne Cédex, France; ‡ Materials Engineering Special Coordination, Universidade Federal de Santa Catarina (UFSC), Blumenau, SC 89036-256,Brazil

## Abstract

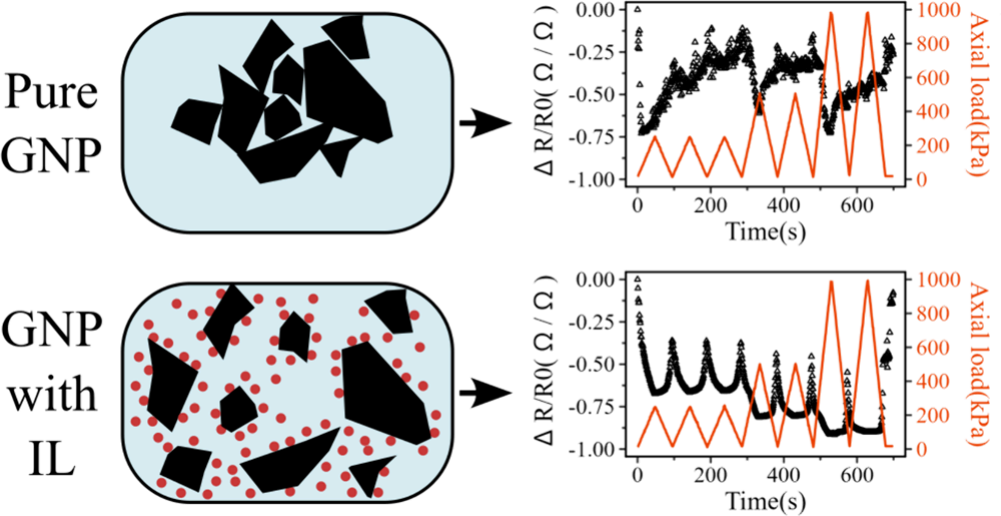

Piezoresistive pressure sensors have been prepared by the electrospinning of poly(vinylidene fluoride) (PVDF) containing graphene nanoplatelets (GNP) functionalized using 1-butyl-3-methylimidazolium trifluoromethanesulfonate (BMIM(OTf)) ionic liquid (IL). Optical microscopy demonstrated that the functionalized GNP powder presented particles with a smaller lateral size. The obtained mats were characterized by scanning electron microscopy, Fourier transform infrared spectroscopy, energy-dispersive X-ray spectroscopy, X-ray diffraction, differential scanning calorimetry, electrical resistivity using two and four probes, and electromechanical testing with up to 32 load–unload cycles. Functionalization with BMIM(OTf) resulted in a higher PVDF electroactive phase. Electrospun mats obtained without the IL displayed a signal comparable to noise, while mats obtained with the BMIM(OTf) functionalized GNP displayed a clear signal, indicating that the IL helped with the dispersion of GNP on the PVDF matrix. Electrospun mats containing 1.0%m functionalized GNP presented the best performance among the evaluated samples, presenting low hysteresis and a lower distribution of the read values especially in the working range of 0 to 250 kPa. The piezoresistive behavior of the sample was tested under 32 load–unload cycles, remaining stable. Higher ranges of axial load resulted in the rupture of the fibers and swift degradation of the piezoresistive signal under a high number of cycles. A simple load cell was assembled to demonstrate the capacity of the membranes to act as piezoresistive compressive sensors capable of detecting the pressing of a human finger and differentiating between applied weights.

## 1 Introduction

Sensing elements represent one of the most significant examples of a measuring device employed daily, receiving a physical stimulus, converting it into an electrical signal that its integrated system can understand, and, in most cases, generating a visual representation of data or starting the actions of an actuator.^1^ The development of new sensors closely follows the development of new electronics, and the demands created by them directly impact the direction of the development of new materials. A material that will compose a sensing element must satisfy the intrinsic needs of the system in which it will be used. Flexible electronics, for instance, require sensors made of materials that result in sensing elements with low dimensions, high energy efficiency, and high flexibility while also ensuring signals with good sensitivity, high linearity, and low hysteresis.^2^

Nanofibers are unidimensional materials in submicrometric scales that present unique properties, including high surface ratio, high porosity, and low density, associated with a high mechanic resistance and flexibility,^3^ leading to a rapid interest in its use as flexible sensing elements,^4^ with recent studies showing the promising performance of nanofibers mats as flexible sensors for strain,^5^ electrochemical,^6^ and optical^7^ sensing. The fabrication of polymeric nanofibers can be done in a simple manner using electrospinning techniques, which consist of applying voltage (commonly in the range from 10 to 20 kV) to a jet of a polymeric solution, by itself or in the presence of fillers, which include materials such as carbon nanotubes (CNTs)^8^ or even other polymers such as polypyrrole.^9^

Among thermoplastic polymers, poly(vinylidene fluoride) (PVDF) presents excellent electroactive properties and a high dielectric constant, making PVDF-based sensors present fast responsivity, an extensive range of operation, high flexibility, and high chemical and strain resistances.^10^ Although studies involving PVDF have focused mainly on its piezoelectricity, the polymer displays good piezoresistive properties with the incorporation of nanofillers.^11^ For instance, Merlini et al.^9^ evaluated the piezoresistive response of electrospun PVDF nanocomposites containing up to 27% polypyrrole nanoparticles, observing a high sensitivity and a high response linearity coupled with a low hysteresis, especially in concentrations of up to 13% polypyrrole.

The piezoresistive effect is defined as a change in the resistivity of the material when submitted to a deformation, where, with the variation of the applied deformation, there is a variation of the resistance value.^12^ Hence, it is possible to directly translate the deformation into electrical signals, allowing those materials to be used as sensing elements.^13^ In a polymer, three main mechanisms govern the piezoresistive behavior: (i) forming of conductive paths by conductive fillers in multiphase systems, (ii) tunneling effects between neighbor fillers, and (iii) the intrinsic piezoresistivity of the polymer and the filler.^14,15^

In the case of nanofibers, the first mechanism of the piezoresistive behavior, with the production of multiphase systems using conductive fillers, proves to be a highly effective way to obtain a piezoresistive material. Due to the high porosity of the fibrous mats, the conductive paths are formed by small sections of fibers containing the conductive fillers, which can be brought into contact with each other by the application of an external pressure even in materials containing low fractions of fillers.^8,9^ Li et al.^16^ obtained a highly sensitive piezoresistive compression sensor by decorating TPU electrospun fibers capable of detecting a series of human motions in a wide pressure range with low and high rates of compression.

A series of conductive materials are being studied to be applied to piezoresistive sensors. For instance, Xing et al.^17^ produced a double-layer self-adhesive tensile-strain wearable sensor made by spraying silver nanoparticles as a sensing layer over a biocompatible polyurethane/d-sorbitol/glycerol adhesive layer. The intimate contact between the materials and the skin gave the sensor enough sensitivity to be able to identify minor movements of the body, such as differentiating between slow and rapid breathing movements and being able to sense the neck pulse. Among conductive materials, carbon-based nanofillers proved to be a highly effective way to obtain a piezoresistive material that can be readily applied as a sensor. Chen et al.^18^ demonstrated a low-cost approach to produce wearable strain sensors by coating a Spandex fiber with a thermoplastic polyurethane (TPU)/multiwalled CNT, resulting in a sensor that could identify movements such as the bending of an index finger and the blinking of a person.

Graphene nanoplatelets (GNPs) are carbon-based materials obtained by the stacking of 10 or more graphene layers, with lateral dimensions commonly between 100 nm and 100 μm,^19^ and present a middle ground between the properties of graphite and those of pure single-layer graphene with an even much greater ease of obtaining than the last one.^20^ Marra et al.,^21^ for instance, developed piezoresistive strain sensors based on water inks containing GNP and deposited on polyester tissues. The resulting material displayed high sensitivity and clear, constant responses. Moreover, Zhang et al.^22^ produced piezoresistive strain sensors made of a polyurethane sponges coated with a combination of GNP and CNT, achieving a high-performance sensor that could be applied as a wearable or even in a sensor array.

However, the efficiency of conductive nanoparticles on polymer nanocomposites depends on the adequate dispersion of the said nanoparticles inside the polymeric matrix. A poor dispersion results in a sensor with low sensitivity and accuracy, besides limiting the range of the generated signal. Nanoparticle agglomeration is a common factor due to high surface energy, minimizing the desirable filler properties and the reproducibility of the manufacturing process.^23,24^ Different strategies of achieving higher degrees of dispersion have been studied, including agitation and sonication of nanoparticles suspended in a liquid medium,^25^ the self-segregation of nanoparticles,^26^ chemical functionalization,^27^ and exploiting noncovalent interactions that can be achieved between the nanoparticles and a functionalizing agent.^28^

The functionalization of nanoparticles using ionic liquids (ILs), inorganic salts with melting points below 100 °C, is one of those strategies and proves to be an efficient way to increase the dispersion of carbonaceous nanofillers thanks to its ability to interact with fillers by noncovalent means through cation−π, cation–cation and van der Waals interactions, encapsulating them and avoiding agglomeration.^20,29^ Residual IL on the filler may also help the dispersion of graphene on the polymer matrix by creating a bridge between the filler and the matrix.^30^

Ruan et al.^31^ prepared IL functionalized graphene using 1-butyl-3-methylimidazolium tetrafluoroborate and successfully incorporated it into a polyimide matrix, obtaining improved dispersion compared to nonfunctionalized graphene and consequently achieving better tribological, mechanical, and thermal properties on the composite. In a similar manner, Dos Santos et al.^32^ achieved a high degree of dispersion of CNT inside a PVDF matrix by the noncovalent functionalization of the nanoparticles using phosphonium ionic liquids. Carbon-based nanoparticles functionalized with ionic liquids have been applied for the manufacturing of piezoresistive sensors. Pan et al.,^33^ for instance, constructed a 3D printed TPU/CNT-IL sensor that achieved high sensitivity and a good resistance. The sensor was applied as a wearable sensor capable of detecting vocal cord vibrations.

With that in mind, we developed PVDF electrospun fibers containing GNP functionalized in a noncovalent method using 1-butyl-3-methylimidazolium trifluoromethanesulfonate (BMIM(OTf)) IL as a way to obtain an electroactive material for use as a pressure sensing element, evaluating the changes on nanofiber mat morphology, electrical conductivity, and piezoresistive properties with the use of different fractions of functionalized GNP and seeking to determine the ideal operating range of the mats.

## 2 Experimental Session

### 2.1 Materials

**Figure 1:**
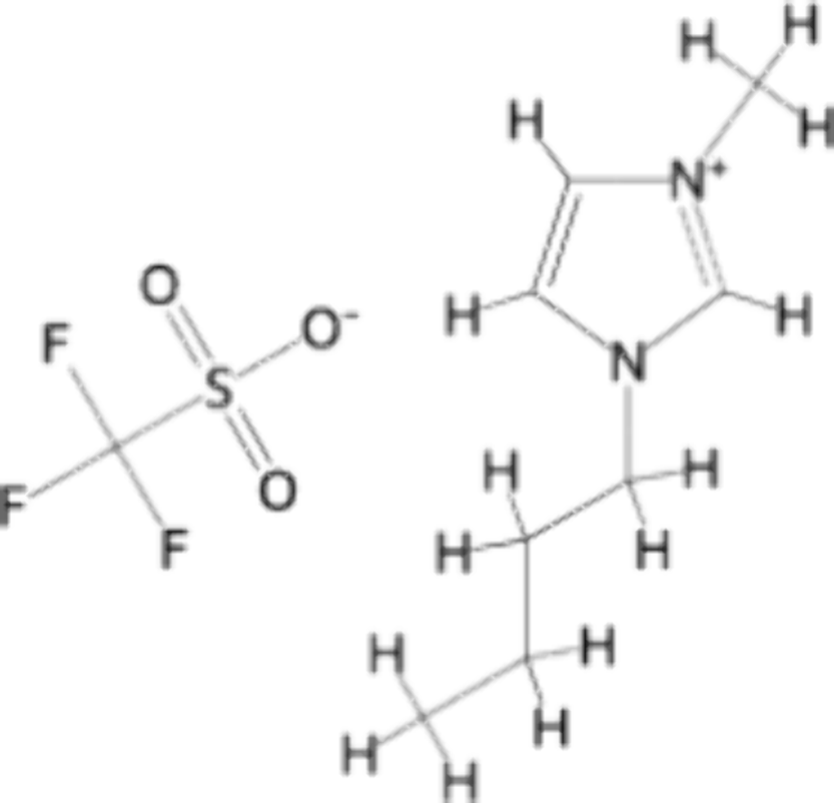
Chemical structure of BMIM(OTf).

For the noncovalent functionalization process of GNP (XG Sciences, M5, 95.1% carbon), *N′N*-dimethylformamide (DMF) (Neon, 99.8%) and acetone (C_3_H_6_O) (Neon, 99.5%) were used, with BMIM(OTf) (Sigma-Aldrich, 98%) as IL. For the electrospinning process, PVDF (Sigma-Aldrich, Mw ∼534,000) was employed as the polymeric matrix and dissolved in a solution of DMF and C_3_H_6_O. Figure 1 presents the chemical structure of BMIM(OTf).

### 2.2 GNP Noncovalent Functionalization Using BMIM(OTf)

The GNP noncovalent functionalization was performed by grinding the particles using mortar and pestle for 20 min in a solution of DMF/C_3_H_6_O 60:40 containing BMIM(OTf), forming a paste. To avoid discrepancies caused by grinding, we employed the same process for samples without IL.

### 2.3 Electrospinning of the PVDF-GNP/BMIM(OTf) Membranes

Electrospun solutions have been prepared by adding 20%m PVDF to the GNP/BMIM(OTf) paste as a function of the sum of the DMF and C_3_H_6_O weights, as described by Merlini.^15^ The solutions were stirred for 4 h at 70 °C under reflux. The resulting GNP suspension was further dispersed using a 1/2″ ultrasonic probe (SONICS Vibra-Cell, model VCX 750) at 200 W for 30 min divided into 5 min periods with a 1 min break between them. The suspensions were spun for 1 h using a needle with an internal diameter of 0.8 mm with a needle/collector distance of 15 cm and coupled with a syringe pump at a flow rate of 2 mL·h^–1^. A high voltage supply generated the electric field applying voltages between 12 and 17 kV. The differences in voltage were necessary to ensure jet stability even with variations between the ionic conductivity and viscosity of the suspensions due to the varying concentrations of graphene and IL. Therefore, pure PVDF samples were spun applying a 12 kV voltage, PVDF/BMIM(OTf) samples were spun applying a 16 kV voltage, and all samples containing GNP were spun applying a 17 kV voltage. Fibers were collected on an aluminum foil at an average ambient temperature of 23 °C and an average relative humidity of 60%. Ten samples were made with different weight fractions of GNP and BMIM(OTf) (both as a function of the PVDF weight on the solution), as established in Table 1.

**Table 1:** Sample Nomenclature and Respective Weight Fractions of GNP and BMIM(OTf) as a Function of PVDF Weight

**sample**	**%m GNP**	**%m BMIM(OTf)**
PVDF	0.0	0.0
0.5GNP	0.5	0.0
1.0GNP	1.0	0.0
1.5GNP	1.5	0.0
2.0GNP	2.0	0.0
PVDF + BMIM	0.0	2.0
0.5GNP + BMIM	0.5	2.0
1.0GNP + BMIM	1.0	2.0
1.5GNP + BMIM	1.5	2.0
2.0GNP + BMIM	2.0	2.0

### 2.4 Characterization Techniques

To visualize the effects of the BMIM(OTf) and the grind process over the GNP particles, the ground GNP, with and without IL (GNP/BMIM(OTF), 1:1), was dried and dispersed in isopropanol using an ultrasonic bath (UNIQUE, model USC-1400) at 40 kHz for 10 min at a concentration of 0.1 mg/mL. Ten microliters of the resulting dispersion was added over a heated glass slide, which was then dried in a laboratory oven at 40 °C for 4 days. A polarized light microscope (Olympus, model DP73) was used to visualize the resulting samples. The lateral size of the GNP particles was measured using the ImageJ software by the methodology described by Pollard et al.^34^

Before electrospinning, the ionic conductivity of the polymer solutions was measured to better understand the effects of the IL in the process. Measurements were made using a conductivity meter (Tecnopon, model mCA 150) with a reference temperature of 20 °C.

Fiber morphology was evaluated by scanning electron microscopy (SEM) (JEOL, model JSM-6390LV). Samples were covered in 100 Å of gold by vapor deposition and scanned at a 10 kV accelerating voltage. The resulting micrographs were used to calculate the nanofiber diameters using the ImageJ software with 100 measurements taken on random points transverse to fiber length.

Energy-dispersive X-ray spectroscopy (Philips, model 30) area scans were employed to determine the presence of residual IL on the surface of the samples, both in the general sample and in areas of the sample, which consist mainly of GNP nanoparticles.

Attenuated-total-reflectance Fourier transform infrared spectroscopy (FTIR) (PerkinElmer, model Frontier FTIR) has been employed in the range from 400 to 4000 cm^–1^, accumulating 16 scans at a resolution of 8 cm^–1^.

X-ray diffraction (XRD) (Malvern Panalytical, model X̀pert) patterns were collected with a Cu Kα source (λ equal to 0.15148 nm) at a 45 kV voltage and 30 mA current and operating in the 2θ range from 5 to 30° at 0.1°·s^–1^.

The transition temperatures and the crystallinity of the matrix were evaluated by differential scanning calorimetry (DSC) (PerkinElmer, model Jade-DSC) under a nitrogen atmosphere at a flow rate of 50 mL·min^–1^ and a heating and cooling cycle of 40 to 220 °C at a rate of 10 °C·min^–1^. The percent crystallinity was obtained through eq 1, where Δ*H*_f_ is the fusion enthalpy of the sample, Δ*H*_f_* is the fusion enthalpy of a perfectly crystalline PVDF (104.5 J·g^–1^^35^), and φ is the weight fraction of PVDF in the samples (between 100 and 96%, according to the sample).
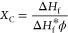
1

Electrical conductivity data were obtained using two- and four-probe methods.^36^ The two-probe method (induce voltage, read current) was used to measure the electrical resistivity of the samples without GNP using an electrometer with a built-in voltage source (Keithley, model 6517A) and a dual-probe high resistivity measuring apparatus (Keithley, model 8009). The volumetric resistivity (ρ) in Ω·cm^–1^ by two probes is given by eq 2, where *d* is the diameter of the electrode supporting the sample, *g* is the distance between the electrode and the outer security ring, *w* is the sample thickness, *V* is the applied voltage, and *I* is the measured electrical current.
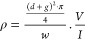
2

Similarly, the four-probe method was used to measure the electrical resistivity of the samples containing GNP (induce current, read voltage) using a source and measure unit (Keithley, model 2410) as a current source and an electrometer (Keithley, model 6517A) as a separate reading unit. The resistivity was measured in three points on both sides of each sample as a way of evaluating the uniformity of the GNP dispersion. In this method, ρ is given by eq 3.
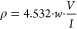
3

Once the resistivity values are obtained, we calculate the conductivity (σ) as the inverse of ρ, as exhibited in eq 4.
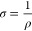
4

**Figure 2:**
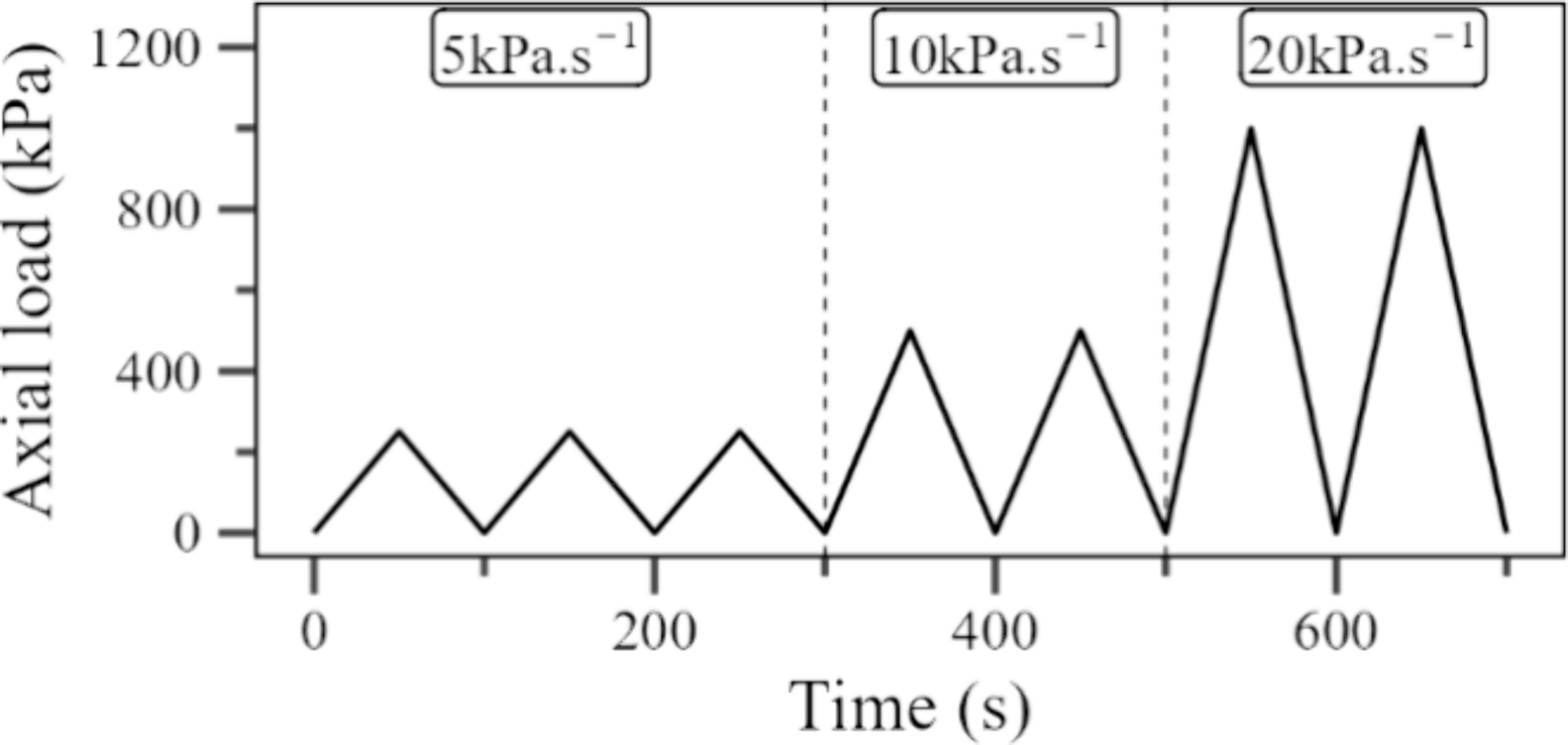
Axial load profile as a function of the running time of the electromechanical tests. The axial load in kPa·s^–1^ is displayed for every cycle.

The electromechanical response of the electrospun mats was evaluated using a universal test machine (INSTRON, model 23-100) for applying a controlled compression on the sample and an electrometer (Keithley, model 6517A) for measuring the changes in the electrical resistivity.^15^ The samples were put between two copper electrodes (diameter of 22.5 mm) connected to the electrometer and confined in a poly(tetrafluoroethylene) cylinder. The sample holder was then placed between the testing plates of the universal machine. Tests were run in seven cycles at a frequency of 0.01 Hz, as shown in Figure 2. Additionally, to evaluate the behavior of the samples under a high number of load–unload cycles, electromechanical assays were performed applying 32 cycles of the ideal operating range observed for each sample.

Data were collected using a software developed exclusively for the electromechanical tests. The variation in resistance Δ*R*/*R*_0_ for each point is then calculated by eq 5, where *R* is the measured electrical resistance for that point and *R*_0_ is the initial electrical resistance measured by the equipment.
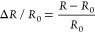
5

As a way of measuring the piezoresistive performance of the samples as compressive sensors, the pressure sensitivity (PS) was calculated, as shown in eq 6, where *P* is the applied axial load in kPa.
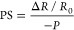
6

## 3 Results and Discussion

### 3.1 Observed Changes in the Dispersion of the GNP Particles

**Figure 3:**
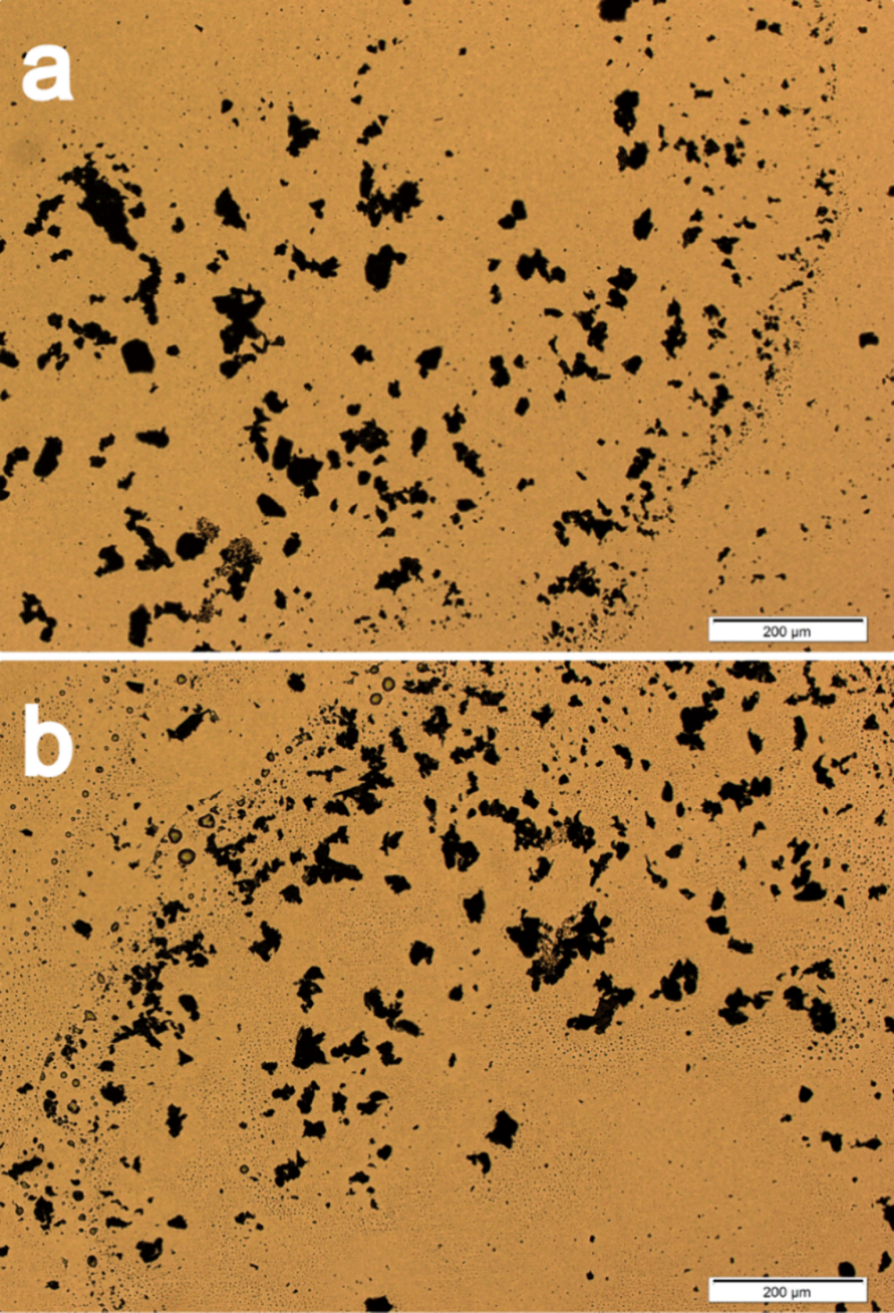
Optical microscopy images of the GNP powders ground (a) without and (b) with BMIM(OTf).

Figure 3 presents the optical microscopy images of the GNP powders ground with and without BMIM(OTf), while Table 2 presents the average lateral size and standard deviation of the particles. The GNP functionalization with the IL resulted in a reduction of approximately 16% of the particles lateral size, as well as a decrease in the standard deviation, characterizing a greater homogeneity in particle size with the use of the IL. The results indicate the interaction of the BMIM(OTf) with the GNP particles, encapsulating them and hindering their agglomeration.^20,37^

**Table 2:** Average Lateral Size of the GNP Samples Ground with and without BMIM(OTf)

**sample**	**average lateral size of the GNP (nm)**
GNP	705.93 ± 1012.90
GNP + BMIM(OTf)	591.10 ± 765.01

**Figure 4:**
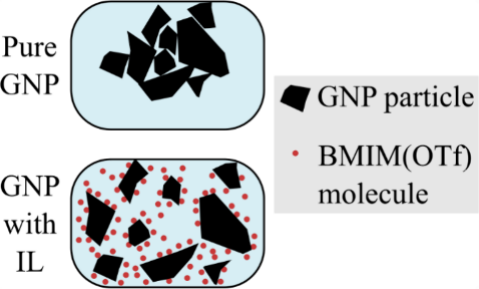
Diagram demonstrating the BMIM(OTf) effect over the GNP particles in suspension through noncovalent functionalization.

The diagram shown in Figure 4 presents the effect of the IL over the macrodispersion of the GNP particles in a suspension. The noncovalent interactions between the particles and the BMIM(OTf) result in an IL barrier that hinders the agglomeration of the GNP particles, increasing dispersion and helping stabilize the suspension.^20,31,32,38^

### 3.2 Observed Changes in the Ionic Conductivity of the Solutions

**Figure 5:**
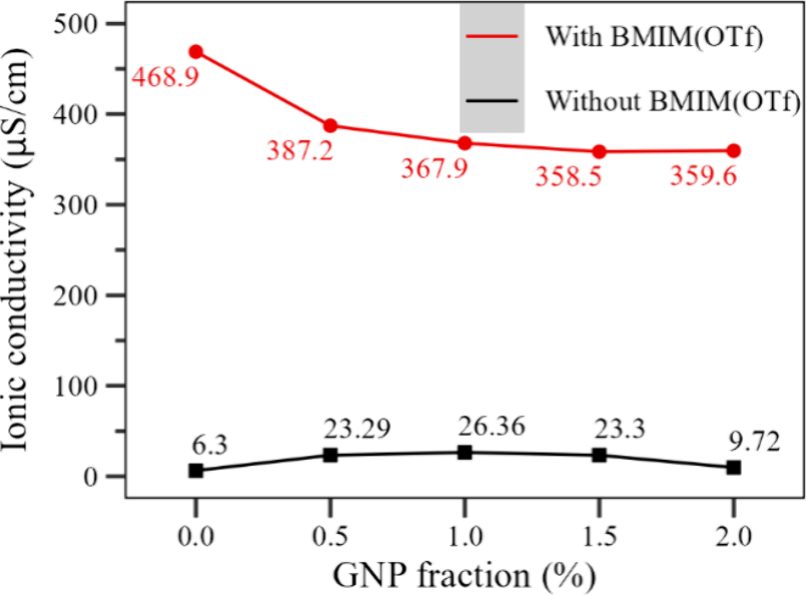
Ionic conductivity of the electrospun solutions as a function of the GNP fraction. Black line: samples without BMIM(OTf); red line: samples containing BMIM(OTf).

Figure 5 presents the measurements of the ionic conductivities of the polymer solutions at each GNP fraction without and with the presence of BMIM(OTf). For the solutions made without the IL (black line), there is a slight increase in the ionic conductivity of the solutions up to until 1.0%m, after which there is a decrease in the values, indicating a higher fraction of GNP agglomerates in solution. The use of BMIM(OTf) (red line) caused a significant increase in the ionic conductivity, with the solutions containing the IL displaying values 20 times higher than those made without the IL, a consequence of the free ionic charges that are now present in the solutions. The addition of GNP, however, caused a decrease in the conductivities of the solutions, indicating an interaction between the nanoparticles and the IL, reducing the quantities of free charges.^20,39^

### 3.3 Observed Changes in the Morphology of the Fibers

**Figure 6:**
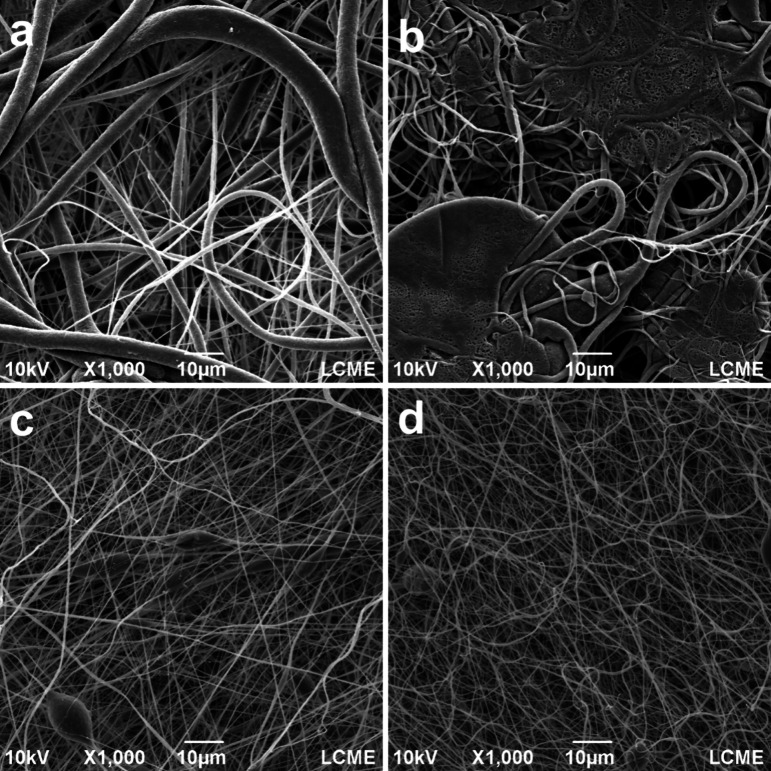
SEM micrographs of the following samples: (a) PVDF, (b) PVDF + BMIM, (c) 2.0GNP, and (d) 2.0GNP + BMIM.

Figure 6 presents the SEM micrographs of electrospun fiber mats made of (a) pure PVDF and with (b) 2.0%m BMIM(OTf), (c) 2.0%m GNP, and (d) 2.0%m GNP functionalized with BMIM(OTf). Table 3 presents the average diameter of the fibers measured from the SEM micrographs.

**Table 3:** Mean Diameter of the Fibers as a Function of the GNP and BMIM(OTf) Fractions on Each Sample

**%m GNP**	**%m BMIM(OTf)**	**average diameter of the fibers (nm)**
0.0	0.0	1840.13 ± 2078.59
0.5	0.0	343.21 ± 172.54
1.0	0.0	337.82 ± 152.93
1.5	0.0	272.25 ± 190.03
2.0	0.0	243.22 ± 156.39
0.0	2.0	896.85 ± 584.60
0.5	2.0	294.92 ± 160.93
1.0	2.0	404.17 ± 253.36
1.5	2.0	558.63 ± 249.10
2.0	2.0	253.76 ± 142.36

Based on the assembled data, it is possible to observe the differences in the formation of the electrospun fibers on each sample: For the sample with pure PVDF (a), the resulting mat is formed by fibers with a high diameter and standard deviation (average diameter of 1840.13 ± 2078.59 nm). The addition of BMIM(OTf) (b) causes a considerable decrease in fiber diameter (average diameter of 896.85 ± 584.60 nm) due to the increment in the stretching of the solution during electrospinning. However, a high defect density was observed in the fibers, signaling inadequate solvent evaporation. This inadequate evaporation can be explained by the high instability of the polymeric jet caused by the excess of BMIM(OTf) ions in the solution, as indicated by the ionic conductivity analysis (Figure 5), which causes a higher interaction between the solution and the electromagnetic field during the electrospinning process, accelerating the polymeric jet. With no means of stabilization, the fibers are collected too early, leading to the high defect density observed.^40,41^

On the samples made with pure GNP (c), there was a more significant reduction in the average fiber diameter (average diameter of 299.13 ± 173.34 nm) but coupled with a low defect density. The addition of the nanoparticles resulted in an increase of the electrical conductivity of the solution, same as with the addition of the IL, leading to an increase in the stretching of the jet, reducing the diameter.^42^ In this case, however, the solvent evaporated in a more controlled manner before touching the collector, avoiding fiber junction and lowering the average diameter and defect density of the fibers.

Finally, for the electrospun mats containing the BMIM(OTf) functionalized GNP (d), fibers were formed in a higher average diameter compared to fibers formed using pure GNP (average diameter of 377.87 ± 238.17 nm) but still a lower diameter compared to fibers formed without GNP. The resulting fibers were also more uniform than those observed in the mats made with pure PVDF or containing only BMIM(OTf). This results from the higher jet instability caused by the BMIM(OTf) ions, but, in that case, the interactions between the IL ions and the GNP particles avoid the excess ions in the solution, preventing the formation of defects. Xing et al.^43^ observed a similar effect on PVDF electrospun nanofibers made using 1-butyl-3-methylimidazolium hexafluorophosphate IL.

### 3.4 Observed Changes in the Surface Chemistry of the Nanofibers

**Figure 7:**
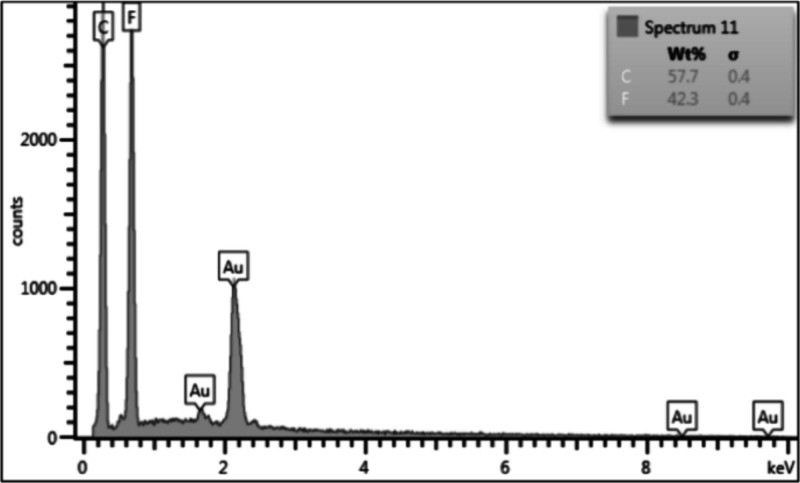
EDS spectrograph, including the read peaks and calculated quantities of each element, for the PVDF electrospun membrane containing 1.0%m GNP functionalized with 2.0%m BMIM(OTf).

Figure 7 presents the EDS spectrograph of the PVDF sample containing 1.0%m functionalized GNP, with other samples presenting similar results. The EDS analysis did not detect the presence of residual BMIM(OTf) on the surface of the samples, on areas either with or without large GNP particles, indicating that the IL was firmly integrated into the nanofiller and into the polymer matrix. This interaction is essential to assist the dispersion of the nanoparticles through the encapsulation of the GNP with the IL, avoiding the formation of agglomerates.^20^

### 3.5 Observed Changes in the Crystalline Structure of the Nanofibers

Regarding the crystalline structures of the materials, the results obtained by FTIR, XDR, and DSC analyses were mutually complementary, with each analysis backing similar points for each change in the microstructure of the fibers.

**Figure 8:**
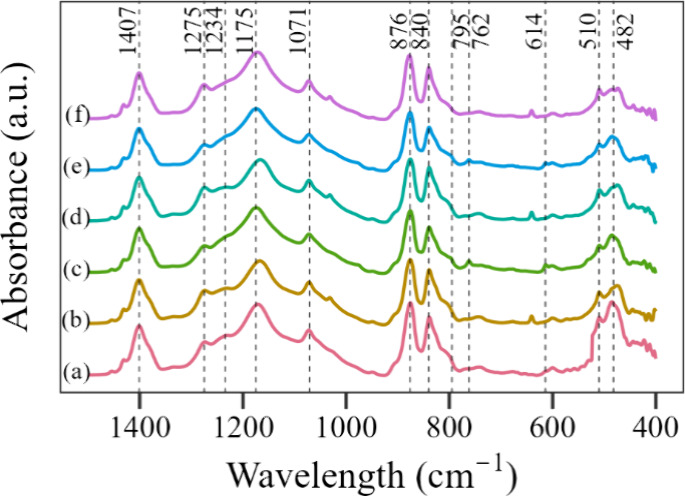
FTIR spectra of the electrospun mats including the PVDF absorption peaks for the following samples: (a) PVDF, (b) PVDF + BMIM, (c) 1.0GNP, (d) 1.0GNP + BMIM, (e) 2.0GNP, and (f) 2.0GNP + BMIM.

The FTIR spectra for the electrospun PVDF mats are shown on Figure 8, displaying well-defined absorption bands for the three main PVDF phases: (i) the α phase in 482 (CF_2_ group bending), 614 (CF_2_ and CCC groups bending), 762 (CF_2_ and CCC groups bending), and 795 cm^–1^ (CH_2_ group rocking); (ii) the β phase in 510 (CF_2_ group bending), 840 (CH_2_ group rocking and CF_2_ group stretching), 1071, and 1275 cm^–1^; and (iii) the γ phase in 1234 cm^–1^ (CF_2_ group bending). There are also peaks for amorphous PVDF in 876, 1175, and 1407 cm^–1^.^15,44^ The spectra show no considerable change in the location of the absorption bands, indicating no significant chemical interaction between PVDF and the additives.

Regarding the crystalline phase intensity, using pure BMIM(OTf) on the polymeric solution increased the PVDF β phase, which is related to a more intense interaction between the polymeric jet and the electromagnetic field, thanks to a rise in the ionic conductivity of the solutions caused by the IL. The addition of pure GNP caused a decrease in β-phase peaks and an increase in α and γ peaks. As demonstrated by Li et al.,^45^ GNP does not act as a nucleating agent for the formation of the β phase in PVDF but can induce the formation of the α and γ phases when the polymer is made with the use of a solvent such as DMF, resulting in a scenario in which the formation of β-phase PVDF is hindered in favor of the formation of α and γ phases. Lastly, the use of the BMIM(OTf) functionalized GNP resulted in an increase in β peaks coupled with a decrease in the intensity of the other phases. As in the mat made with pure BMIM(OTf), that can be explained by the more intense interaction between the polymeric jet and the electromagnetic field.

**Figure 9:**
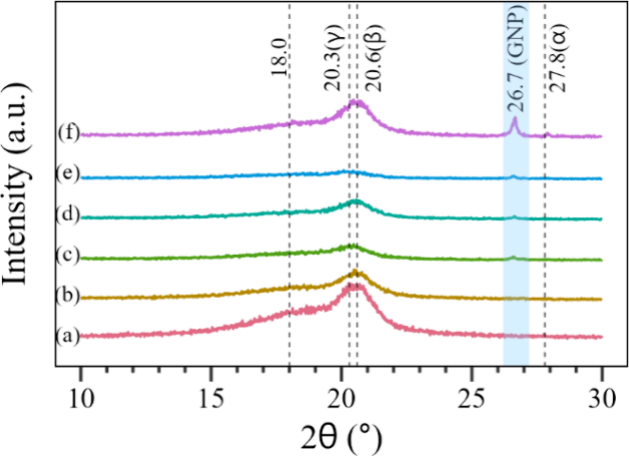
XRD diffractograms, including the PVDF and GNP peaks, for the following samples: (a) PVDF, (b) PVDF + BMIM, (c) 0.5GNP, (d) 0.5GNP + BMIM, (e) 2.0GNP, and (f) 2.0GNP + BMIM.

The XRD diffractograms in Figure 9 display peaks related to the following PVDF planes: (020) from the α phase at 18.0°, (101) from the γ phase at 20.3°, (110) from the β phase at 20.6°, and (111) from the α phase at 27.8°.^15,46^ The (002) GNP plane is also present at 26.7°.^47^ Reinforcing the trend shown by the FTIR spectra, adding pure GNP decreases the electrospun PVDF β phase. It is also possible to notice a decrease in the general crystallinity of the materials. On the other hand, functionalizing the GNP with BMIM(OTf) enhances the β-phase formation and restores part of the PVDF crystalline fraction.

**Figure 10:**
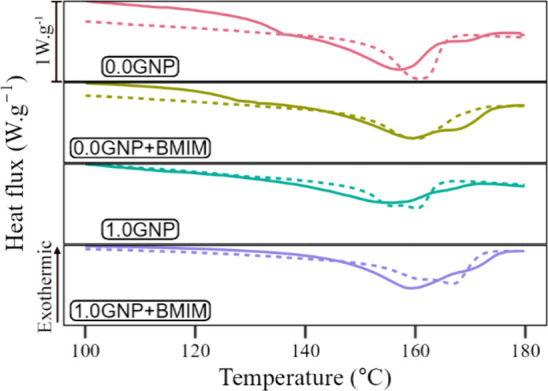
DSC thermograms for the following samples: (red lines) PVDF; (dark yellow lines) PVDF + BMIM; (green lines) 1.0GNP; and (purple lines) 1.0GNP + BMIM. The (continuous line) 1° heating cycle and the (dotted line) 2° heating cycle for each sample are portrayed.

Figure 10 presents the DSC thermograms for the electrospun mats, while Table 4 displays the obtained data for the 1° heating cycle, including the melting temperature (*T*_m_), fusion enthalpy (Δ*H*_f_), and PVDF crystallinity (χ_c_). In the pure PVDF sample (0.0GNP), it is possible to observe an endothermic peak at 156.96 °C attributed to the melting temperature. *T*_m_ is shifted up to 159.20 °C by the addition of BMIM but decreases to 154.04 °C with the addition of pure GNP. The addition of the GNP functionalized with BMIM(OTf) brings the *T*_m_ up again to 159.13 °C.

**Table 4:** Results of the 1° DSC Heating Cycle for the Following Samples: PVDF, PVDF + BMIM, 1.0GNP, and 1.0GNP + BMIM

**sample**	*T*_**m**_**(°C)**	**Δ***H*_**f**_**(J·g**^**–1**^**)**	**χ**_**C**_**(%)**
PVDF	156.96	66.22	63.37
PVDF + BMIM	159.20	55.42	54.12
1.0GNP	154.04	28.61	27.65
1.0GNP + BMIM	159.13	53.13	52.41

The peaks in the thermograms and the values in Table 4 confirm the behavior brought by the XRD diffractograms in Figure 9. There are a substantial decrease in fiber crystallinity by adding pure GNP (χ_c_ = 63% for pure PVDF mat and χ_c_ = 27% for the PVDF mat with 1.0% GNP) and a recovery of this crystalline fraction with the GNP functionalization with BMIM(OTf) (χ_c_ = 52% for the PVDF mat with 1.0%m GNP functionalized with 2.0%m BMIM(OTf)). This behavior is due to two concurrent tendencies: (i) a reduction in crystallinity with the increase of the GNP fraction and (ii) a recovery in crystallinity by functionalizing the nanoparticles with BMIM(OTf). The first tendency (a decrease in crystallinity) occurs by the action of the GNP nanoparticles as nucleating agents, inducing the formation of small crystals with a high fraction of defects and reducing the final crystallinity. The second tendency (a recovery in crystallinity using IL) may be related, again, to a more considerable stretching of the nanofibers during electrospinning thanks to the IL and agrees with the SEM (Figure 6) results as well as other studies concerning the PVDF electrospinning with IL.^48,49^ An alternative explanation is a result of GNP encapsulation with BMIM(OTf), which avoids the nucleating action of the nanoparticles. The same effect was observed for PVDF nanocomposites made with multiwalled carbon nanotubes functionalized using 1-vinyl-3-methylimidazolium tetrafluoroborate IL.^50^

### 3.6 Observed Changes in the Electrical Conductivity of the Nanofibers

**Figure 11:**
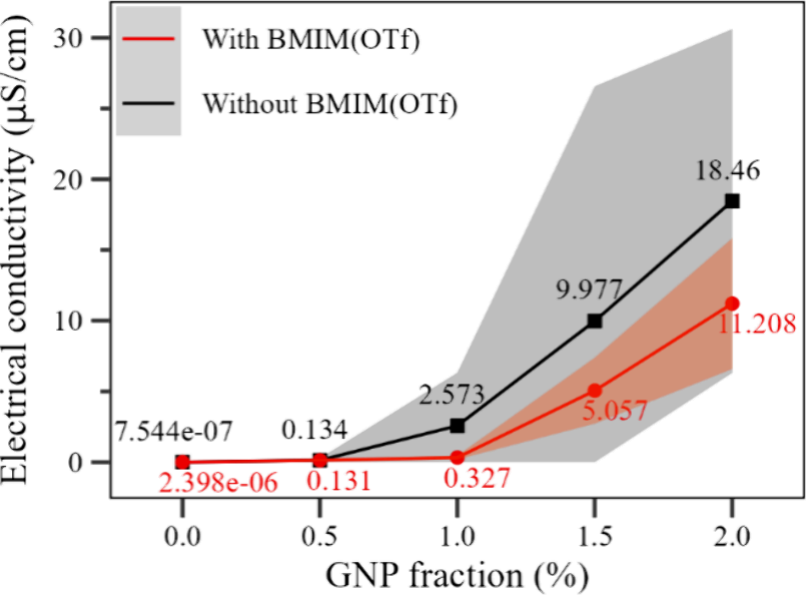
Average electrical conductivity and standard deviation for the PVDF samples as a function of the GNP fraction. Black line: without BMIM(OTf); red line: with BMIM(OTf).

The electrical conductivity of the electrospun PVDF mats has increased significantly with the addition of the GNP nanoparticles, regardless of the presence of BMIM(OTf), as shown in Figure 11. The GNP functionalization with BMIM(OTf) decreased the average electrical conductivity of the mats compared to samples containing an equivalent GNP fraction. That can result from the GNP nanoparticles' encapsulation with IL, which hampers the conducting network formation.^20^ Still, there was a drastic change in the homogeneity of the electrical conductivity measured on samples containing BMIM(OTf).

As the conductivity of the membrane comes from the presence of the GNP filler in discrete points inside the PVDF matrix, as opposed to the continuous conductivity of a pure conductive material, such as a copper wire, the homogeneity of the conductivity values read at different points on each sample can be correlated to the nanoparticle dispersion in the matrix. A higher homogeneity, as observed for the samples containing BMIM(OTf), indicates a better dispersion.

### 3.7 Piezoresistive Response

**Figure 12:**
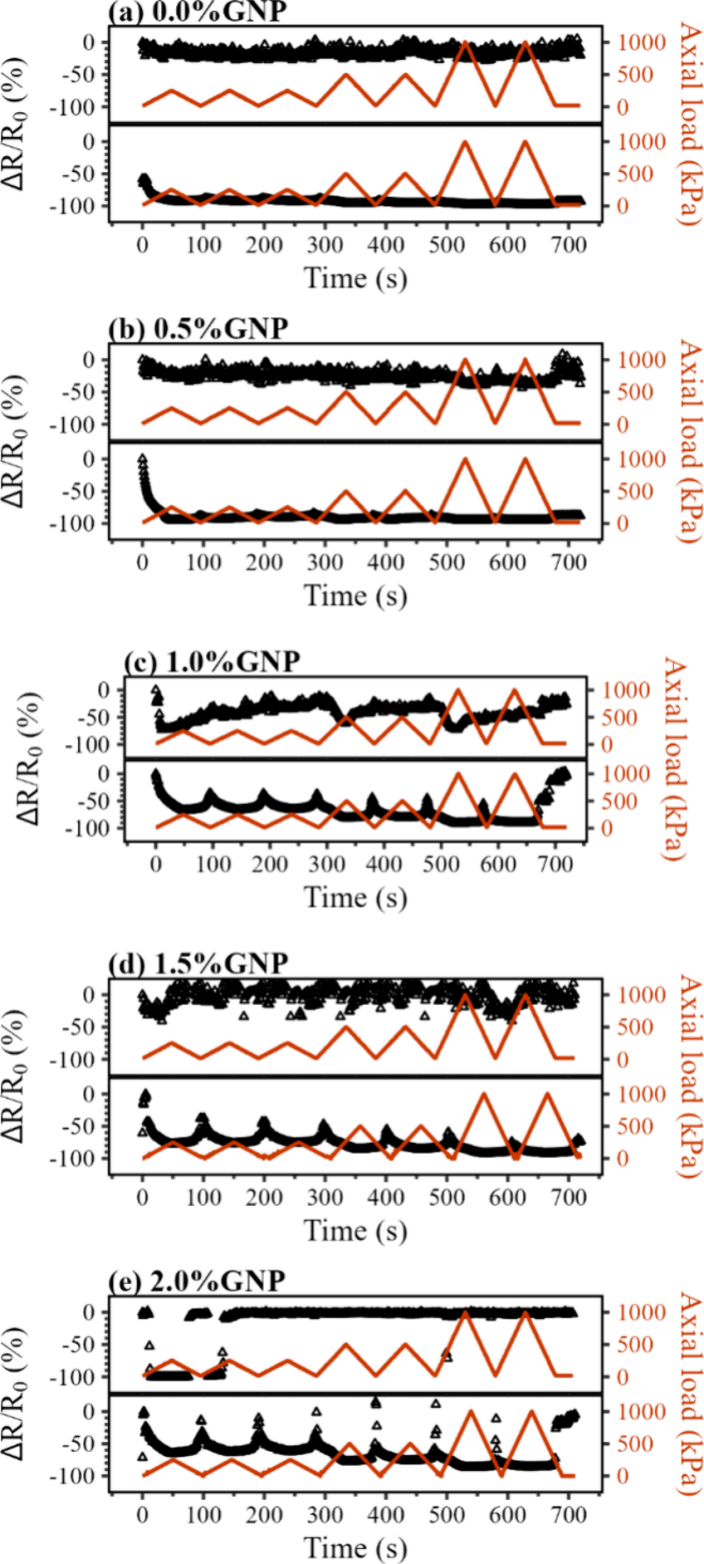
Piezoresistive response (Δ*R*/*R*_0_) (black dots, left axis) and axial load in kPa (red line, right axis) as a function of testing time for the electrospun mats containing (a) pure PVDF and with (b) 0.5%m GNP; (c) 1.0%m GNP; (d) 1.5%m GNP; and (e) 2.0%m GNP. Upper graph: sample without BMIM(OTf). Lower graph: sample functionalized with 2.0%m BMIM(OTf).

Charts presented in Figure 12 allow a comparison between the piezoresistive response of the PVDF mats before and after the GNP functionalization using BMIM(OTf): Samples without BMIM(OTf) displayed responses similar to that of noise, with a high dispersion of the read values and no predictable behavior, and although it is possible to notice valleys on the resulting signal, it is not possible to set a clear behavior, rendering the material unfeasible for use as a sensing element. On the other hand, samples containing IL-functionalized GNP displayed a clear signal with a stable and predictable response. This stability can be explained by a decrease in GNP agglomerate formation inside the nanofibers with the use of BMIM(OTf), which facilitates the formation of conducting paths during the electrospun mat compression, intensifying possible changes in resistivity and lessening the dispersion of values in the signal.

For all of the electrospun mats, the first compression cycle (from 0 to 250 kPa, with no previous deformation) presented a considerably different response from the others, with Δ*R*/*R*_0_ descending rapidly and not returning to its initial values after the end of the cycle. Instead, the read values on the subsequent cycles are confined to a smaller region that grows on higher compression loads. That indicates the need for a predeformation step of the materials before a real-world application, something that can be made on the final sensor assembly.

Regarding samples obtained without GNP (PVDF and PVDF + BMIM), the piezoresistive response was also improved. That occurs due to the BMIM(OTf) itself present inside the fibers, where with the material deformation the IL ions are allowed to move with greater ease, decreasing the electrical resistivity of the material. A similar phenomenon was reported for PVDF thin films containing 1-butyl-3-methylimidazolium chloride IL.^51^

The initial variation in electrical resistance (Δ*R*/*R*_0_) is lowered with an increase in the GNP content on the samples. Simultaneously, the valleys of Δ*R*/*R*_0_ increase, facilitating the piezoresistive response distinction as a function of the axial load. That behavior results from the different ways in which the conductive paths are formed with the varying GNP fractions:

i. With a lower GNP fraction, the conductive paths formed by the particles have a higher impact, making the electrospun mat more effective in detecting lower compressive stress variations, especially on lower ranges. On the other hand, a lower GNP fraction also makes it so that the formation of the conductive paths saturates more quickly, lowering the Δ*R*/*R*_0_ valleys' intensity and making it harder to distinguish compressive stress values on higher ranges. That results in sensors with a higher sensitivity but also a lower range;
ii. With a higher GNP fraction, the scenario is inverted: the initial conductive path formation is not as significant on the resulting signal, but a higher number of conductive paths are formed before the saturation of the mats. That results in sensors with a lower sensitivity on lower axial loads but a higher operating range.

**Figure 13:**
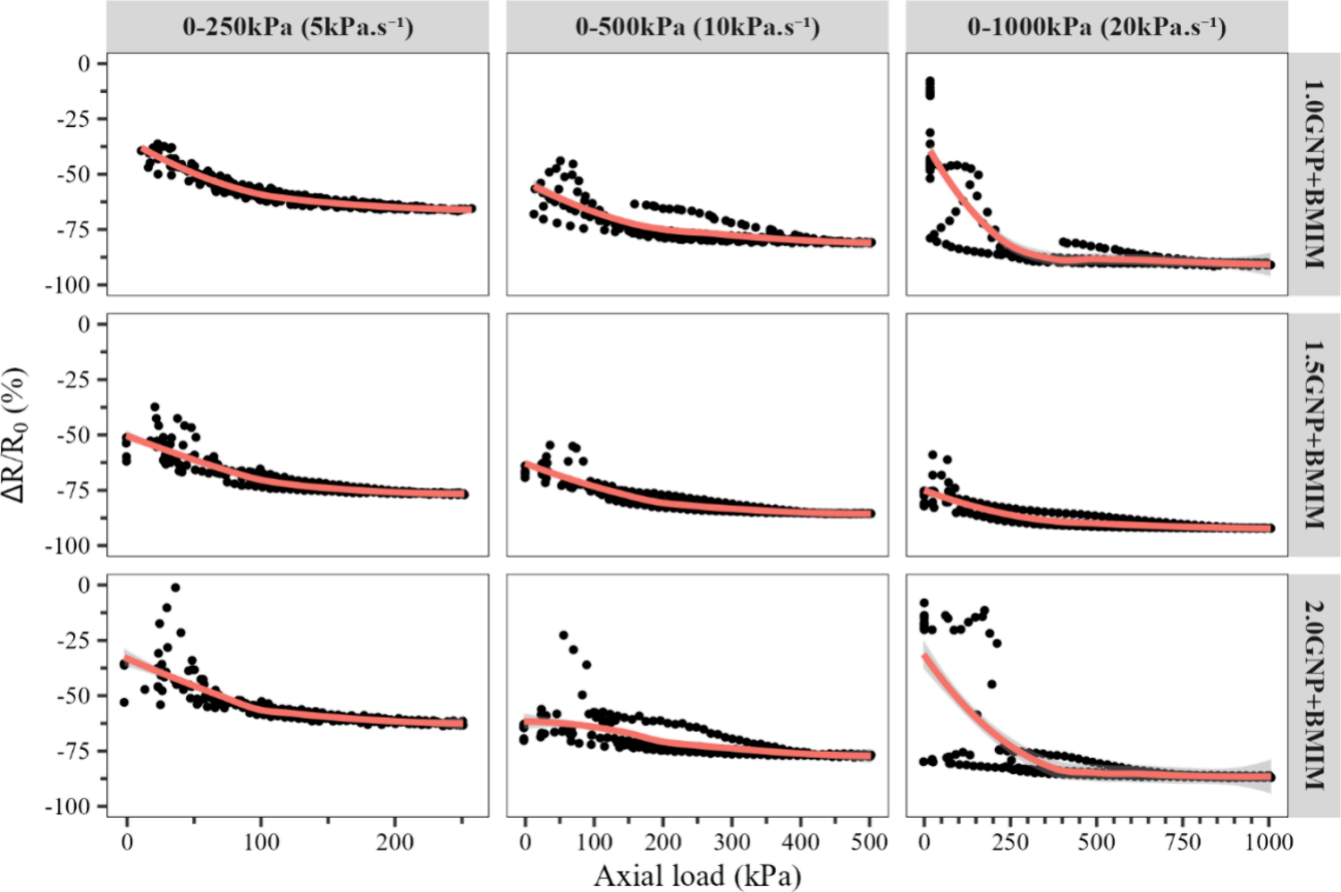
Piezoresistive response (Δ*R*/*R*_0_) as a function of axial load in kPa for the three considered study ranges (0–250, 0–500, and 0–1000 kPa) with the corresponding fitted curves for the following samples: 1.0GNP + BMIM, 1.5GNP + BMIM, and 2.0GNP + BMIM.

Both the sample of pure PVDF containing 2.0%m BMIM(OTf) and the sample with 0.5%m GNP functionalized with 2.0%m BMIM(OTf) presented stable responses, although with a poor Δ*R*/*R*_0_ valley intensity, rendering them unsuitable for use in piezoresistive sensors. The remaining functionalized samples (1.0, 1.5, and 2.0%m GNP functionalized with 2.0%m BMIM(OTf)) presented valleys that are deep enough to allow the correlation of the read signal into a corresponding axial load value. To ascertain the viability and ideal operating range of those three samples, the piezoresistive response was evaluated as a function of the axial load and load rate for the three studied ranges (0–250, 0–500, and 0–1000 kPa at 5, 10, and 20 kPa·s^–1^, respectively), not considering the first compression cycle, and is presented in the graphs in Figure 13 with the corresponding fitted curves for each plot.

Among the samples, the sample containing 1.0%m GNP functionalized using 2.0%m BMIM(OTf) (1.0GNP + BMIM) displayed a low hysteresis at the 0 to 250 kPa range at 5 kPa^–1^ and a curve with a high enough slope to allow a straightforward association of Δ*R*/*R*_0_ values to the applied axial load. The sample, however, displays a considerably high hysteresis at the 0 to 500 kPa range at 10 kPa·s^–1^ and higher. The hysteresis at those ranges indicates a possible damage to the conductive network with the higher axial load and load rates, leading to the breakage of the polymeric matrix^28^ and the slippage of the GNP particles.^52,53^ Other possible factors that may cause the hysteresis in the material are the Mullins effect, with the change in the elastic behavior of the material with the applied loads, as well as the strain softening effect in the polymer matrix, with the rearrangement of the molecular chains to minimize the deformation energy.^28^

The sample with 1.5%m GNP functionalized using 2.0%m BMIM(OTf) (1.5GNP + BMIM) displayed a higher hysteresis in the range from 0 to 250 kPa compared to the previous sample but displayed a satisfactory behavior on the 0 to 500 and 0 to 1000 kPa ranges, making it less sensitive on lower ranges but able to operate in higher ranges.

The sample containing 2.0%m GNP functionalized using 2.0%m BMIM(OTf) (2.0GNP + BMIM) presented an adequate response in the range from 0 to 250 kPa but less adequate responses in higher ranges. The sample also presents a noisier signal than that of the sample containing 1.0%m of the functionalized GNP in the range from 0 to 250 kPa.

The higher GNP fraction at both the samples containing 1.5 and 2.0%m of functionalized GNP reduces the effect of the slippage of the particles over the conductive network, reducing the hysteresis at higher ranges and load rates that can be caused by this, although at a high number of cycles that can still lead to the deterioration of the resulting signal.

The inferior behavior of the samples containing 1.5 and 2.0%m of functionalized GNP, especially in lower axial loads, can be explained by an excess of GNP inside the fibers, which results in a noisier signal and in quick saturation, with Δ*R*/*R*_0_ valleys of lower depth at higher compression values. The excess of GNP is emphasized in the samples containing 2.0%m of functionalized GNP: the viscoelastic behavior of the polymeric matrix may cause the formed conductive paths not to be easily disconnected by the release of the axial load, resulting in the observed hysteresis.

**Figure 14:**
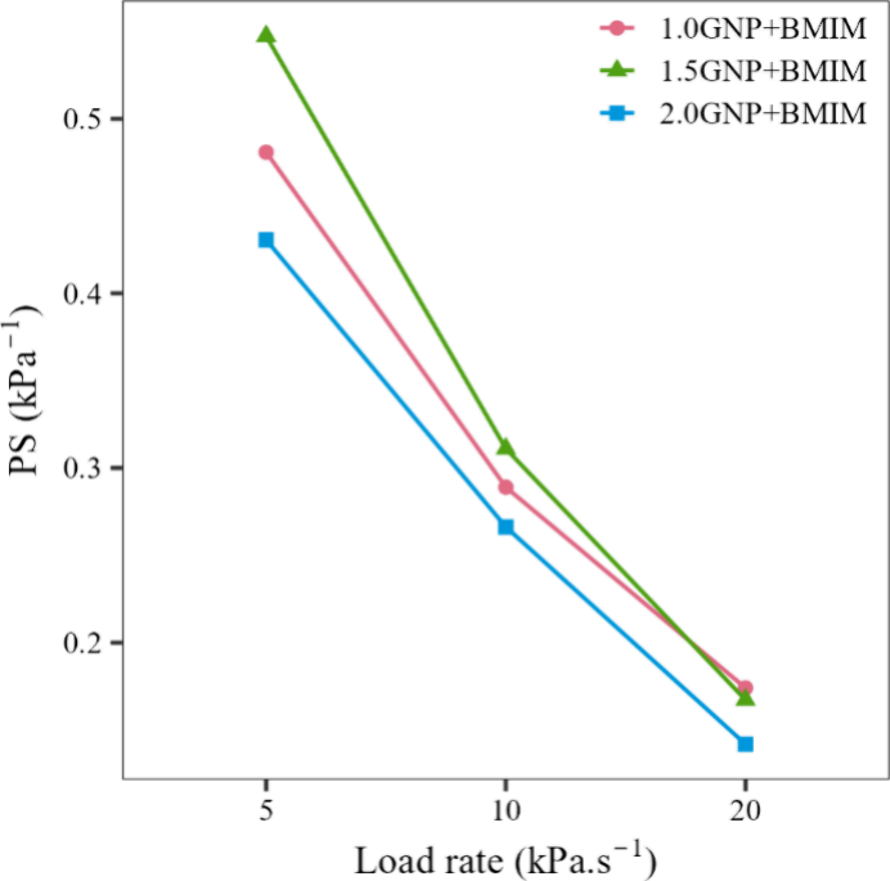
PS in kPa^–1^ as a function of load rate in kPa·s^–1^ for the PVDF samples containing 1.0, 1.5, and 2.0 GNP functionalized with BMIM(OTf).

Figure 14 presents the PS data of the samples at different load rates. In all samples, there is a reduction in sensitivity with increasing load rates. That reduction can be explained by the physical limitation of the polymeric matrix, which leads to a stiffer mechanical response and delays the formation of the conductive network, reducing the sensitivity.^18,28,52^ The values show an increase in the PS values between the samples with 1.0 and 1.5%m of functionalized GNP, a result of the easier formation of the conductive networks with the increase of the filler fraction. The samples containing 2.0%m functionalized GNP, however, presented a lower PS, a direct result of the excess of GNP in the samples, which leads to a fast saturation of the system, reducing the sensitivity of the composite.

Fitting the curves of the piezoresistive signal data allows us to define the ideal operating ranges of each electrospun mat and observe how close the samples are to an ideal sensing behavior. Based on the curves, it is possible to state that the samples with 1.0%m functionalized GNP and 1.5%m functionalized GNP presented the best responses on two distinct operation ranges, with the electrospun mat containing 1.0%m functionalized GNP being ideal in the range from 0 to 250 kPa at 5 kPa·s^–1^ and the sample containing 1.5%m functionalized GNP being ideal in the range from 0 to 500 kPa at 10 kPa·s^–1^, while also being functional in the range from 0 to 1000 kPa at 20 kPa·s^–1^. Both samples presented lower hysteresis and sharper responses in their respective ranges.

**Figure 15:**
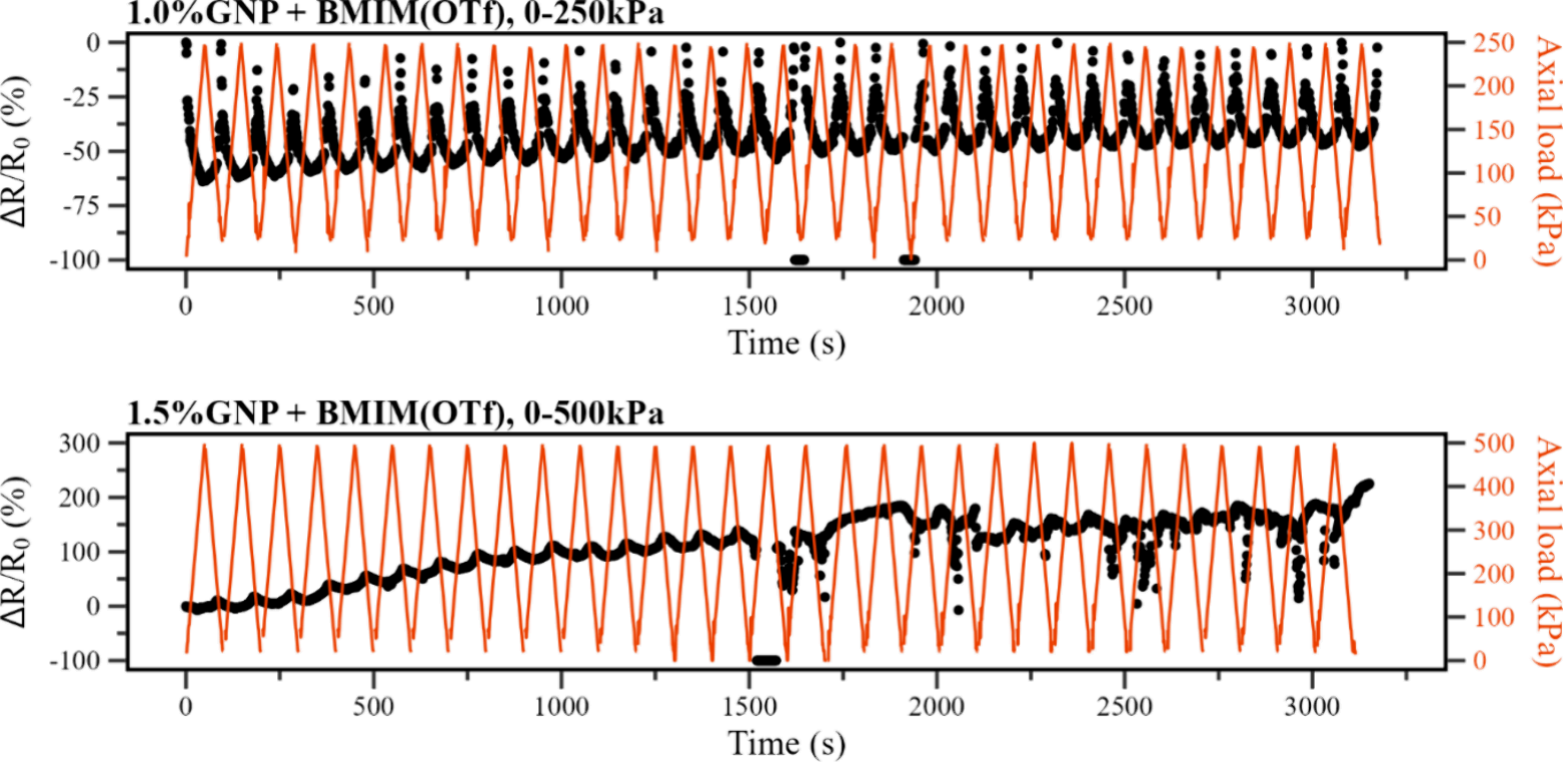
Piezoresistive response (Δ*R*/*R*_0_) (black dots, left axis) and axial load in kPa (red line, right axis) as a function of testing time for the piezoresistive assays made with 32 cycles for the PVDF samples containing 1.0%m GNP + BMIM(OTf) and 1.5%m GNP + BMIM(OTf).

To evaluate the performance of the samples on a higher number of compression cycles, both the samples containing 1.0%m functionalized GNP and 1.5%m functionalized GNP were tested in 32-cycle electromechanical assays under their respective ideal operating ranges. The results presented in Figure 15 demonstrate that, for the sample containing 1.5%m functionalized GNP, despite showing an adequate piezoresistive response on the range from 0 to 500 kPa under a low number of cycles, the sample response quickly degraded under the 32-cycle assay, especially after about 15 cycles, indicating that the electrospun membrane ruptured midway under the test and that the samples are not adequate for use under high compression ranges for a high number of cycles.

The sample containing 1.0%m functionalized GNP, however, maintained a stable response under its ideal operating range from 0 to 250 kPa, with low signal degradation and only a slight Δ*R*/*R*_0_ valley depth decrease on the first eight cycles, which can be caused by the settling of fibers on the initial compression cycles. That settles the PVDF membrane containing 1.0%m GNP functionalized with 2.0%m BMIM(OTf) as the best composition of the evaluated samples for an electrospun piezoresistive sensor element, with an ideal operating range from 0 to 250 kPa.

It is worth noting that the piezoresistive response can be further improved by the design and construction of the sensor as a whole, allowing the shielding of the sensor element to avoid the interference of external noise. Treating the read signals through software may also eliminate noise and further approximate the sensor response to the ideal theoretical response of the fitted curves.

### 3.8 Application of the Electrospun Membrane as a Load Cell

**Figure 16:**
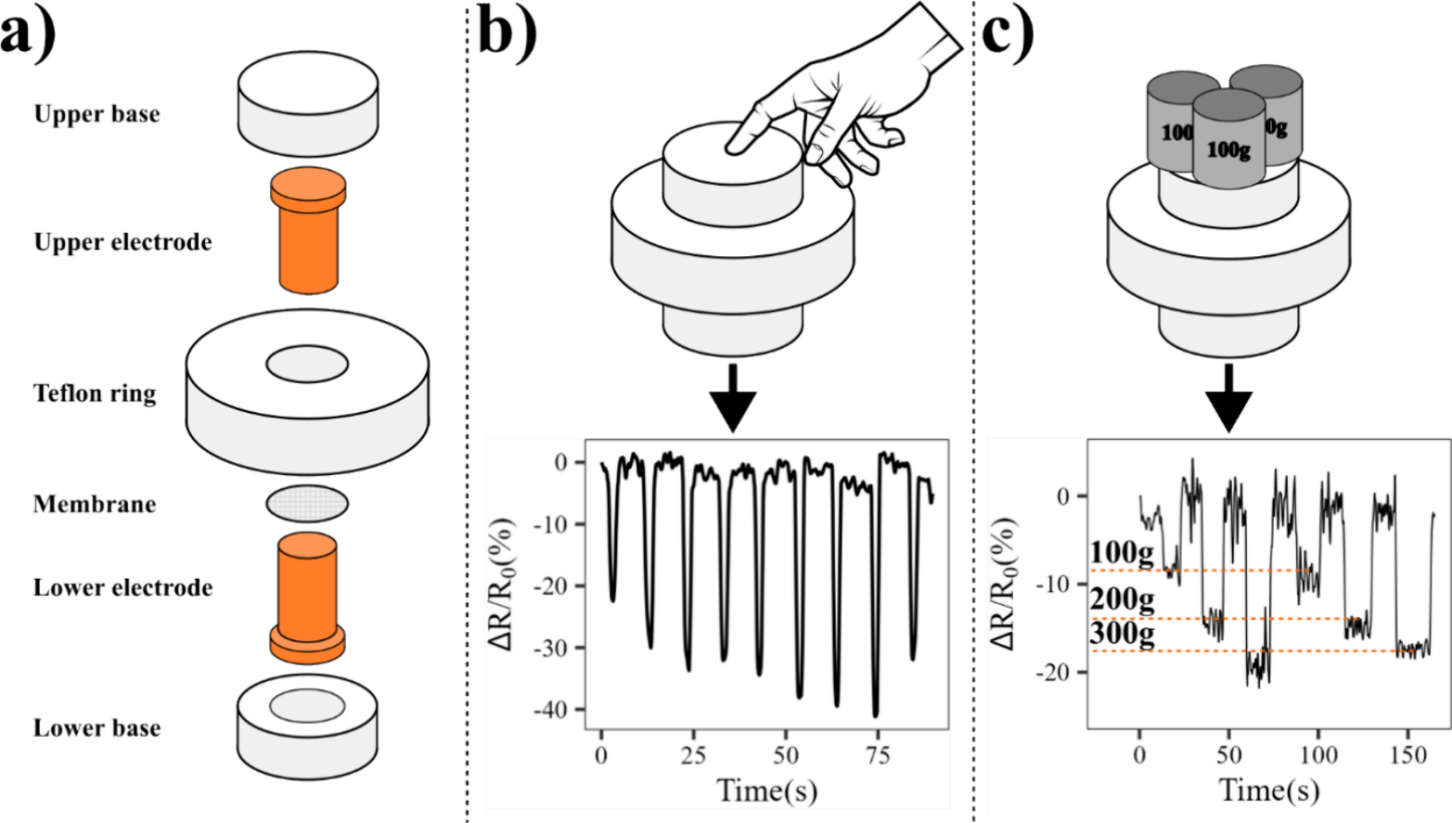
Diagram displaying the application of the membrane containing 1.0%m GNP functionalized with BMIM(OTf) as a load cell. (a) Assembly of the load cell, (b) application of finger pressure over the load cell and the resulting piezoresistive signal, and (c) weights set over the load cell and the resulting piezoresistive signal.

The electrospinning of PVDF fibers containing 1.0%m of functionalized GNP offers an interesting material that can be applied as sensor elements. To exemplify a possible application of the piezoresistive membrane, a simple load cell was assembled, as demonstrated in Figure 16a, with the membrane placed between two copper electrodes that are free to move inside a Teflon chamber. Outside, two Teflon bases isolate the electrodes, which are connected to a circuit that operates at 9 V and reads the variation in the electrical current that flows in the sensor when an axial load is applied to one of the Teflon bases.

In Figure 16b, the load cell is applied to identify the pressing of a finger. The resulting signal can readily identify when the load cell is pressed, and one can easily set an on–off state that is toggled by the pressing of the sensor. Figure 16c demonstrates the use of the load cell as a weight sensor. Three weights measuring approximately 100 g each were placed over the sensor at groups of one, two, and three weights, removing the weights between the groups. The sensor can generate a resulting signal that is different according to the applied weight. Moreover, when the weights are removed, the signal goes back to its original state.

## 4 Conclusions

Electrospun piezoresistive pressure sensors were successfully made using PVDF containing GNP functionalized using BMIM(OTf) with a noncovalent method. The functionalization process resulted in nanofibers with a low defect density and a higher electroactive phase. It was possible to notice a considerable improvement in the piezoresistive response of the electrospun mats with the addition of BMIM(OTf), where it was observed that all of the samples obtained without the IL presented responses similar to that of noise, making them unfeasible to use as a sensing element. On the other hand, mats containing the IL functionalized GNP presented a clear piezoresistive behavior, with the increase in resistivity matching the increase in the axial load.

Among the samples, based on the data obtained during the electromechanical tests, the composition that proved to be the most promising is the one containing 1.0%m functionalized GNP, presenting low hysteresis and a lower response noise on the range from 0 to 250 kPa even under a high number of load–unload cycles and displaying Δ*R*/*R*_0_ values close to those of the fitted, ideal curve. The resulting curve slope was also high enough to allow the association of each Δ*R*/*R*_0_ value to the respective axial load. For higher ranges, although the sample containing 1.5%m GNP presented adequate responses on the ranges of 0 to 500 and 0 to 1000 kPa under a low number of cycles, the electrospun mats do not seem to hold the high axial forces of ranges of 500 kPa and above under a high number of load–unload cycles, resulting in the rupture of the fibers and quick degradation of the resulting signal.

The behavior of the samples displays the efficiency of the BMIM(OTf) functionalization as a means to enhance the piezoresistive properties of electrospun PVDF/GNP nanofibers, allowing their use as pressure-sensing elements, especially when operating in low compression ranges.

The assembled load cell illustrates a possible application of the composite, being able to readily detect the pressing of a finger and differentiate between applied weights. Further applications of similar piezoresistive sensors can include the use of the membranes as flexible wearable sensors for measuring human motion, sensor arrays assembled to detect the precise point where a pressure is applied, and vibration sensors.
